# Supplementary figures and images for: Rapid Test for Adulteration of Fritillaria Thunbergii in Fritillaria Cirrhosa by Laser-Induced Breakdown Spectroscopy
Source: Foods. 2023 Apr 20;12(8):1710. doi: 10.3390/foods12081710 (PMC10138139; doi:10.3390/foods12081710)

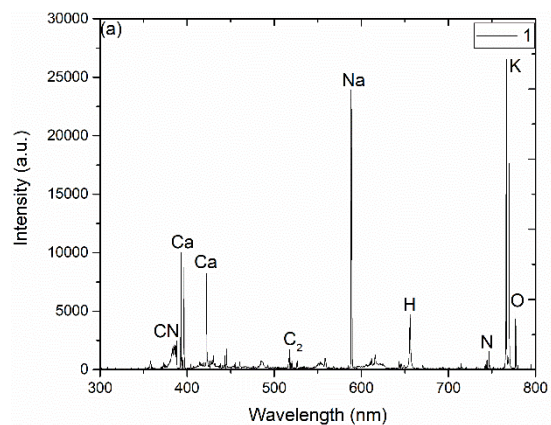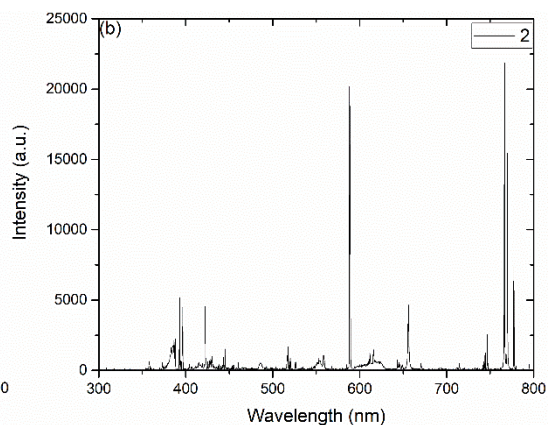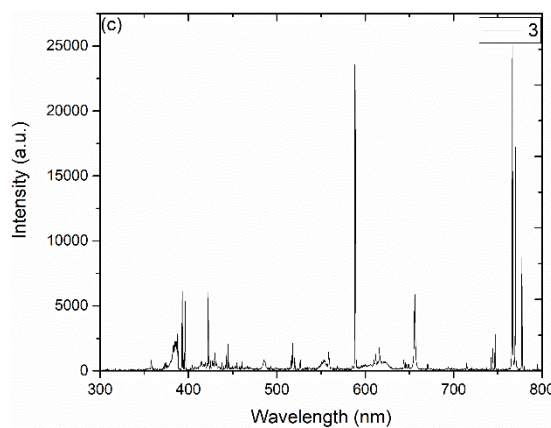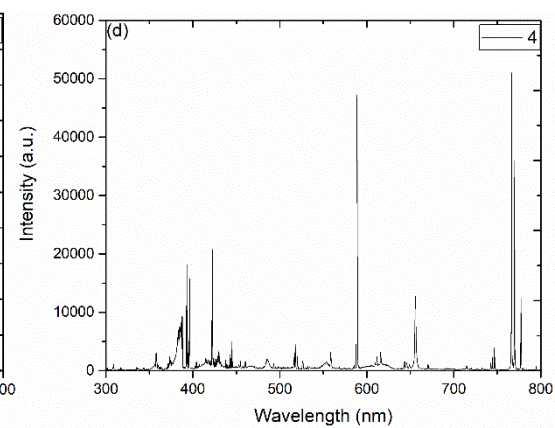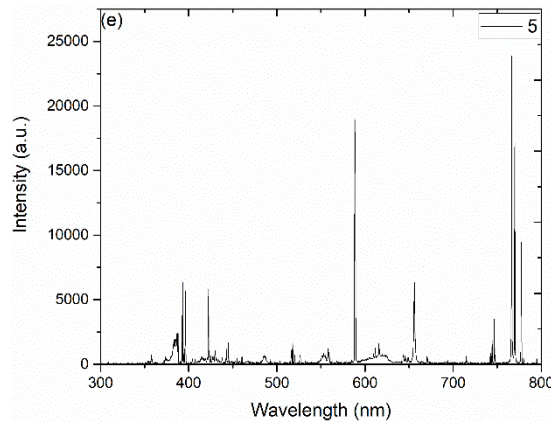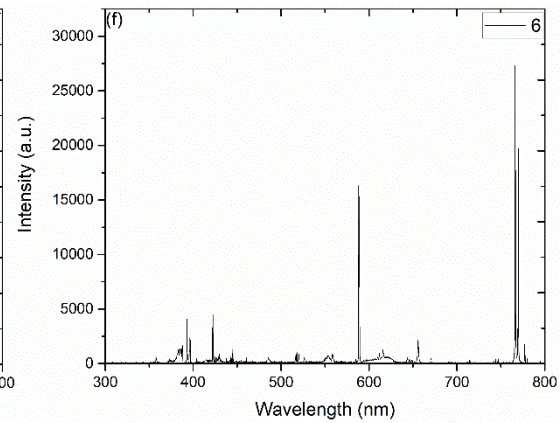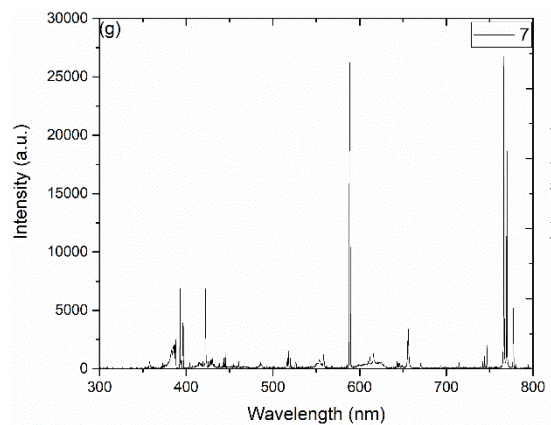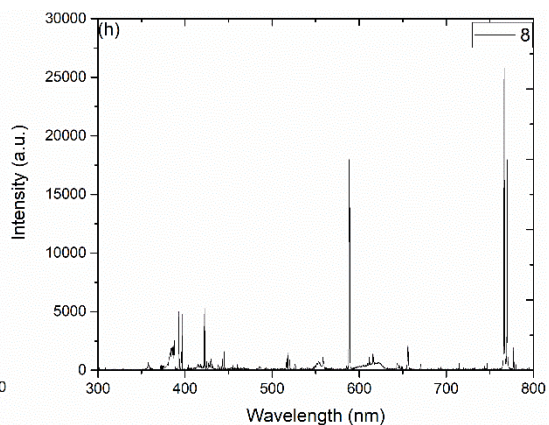

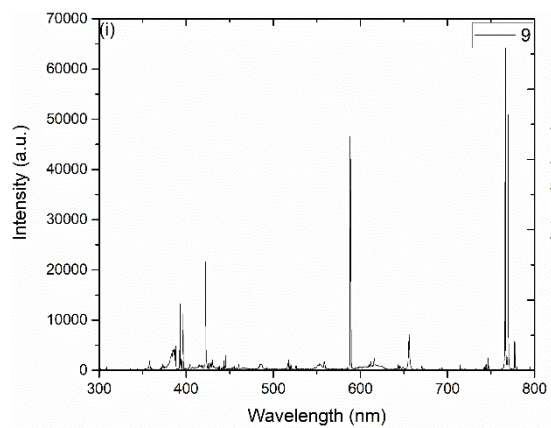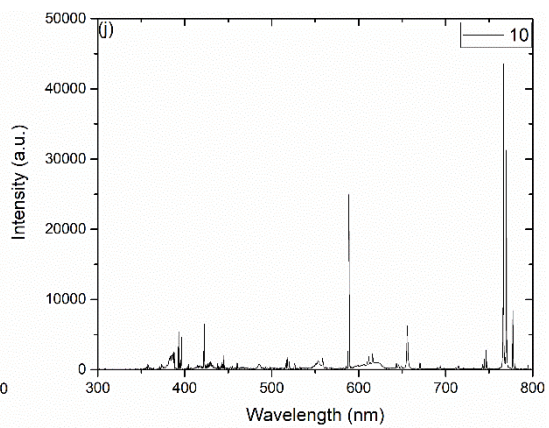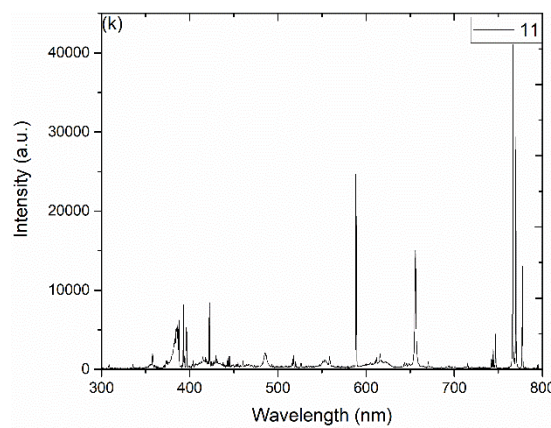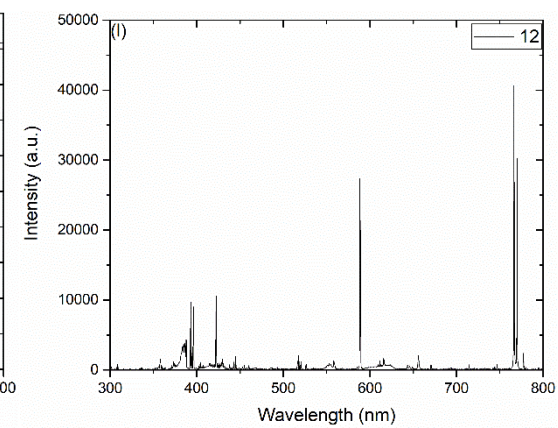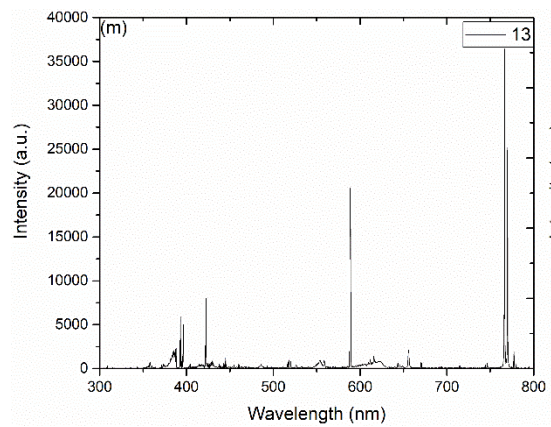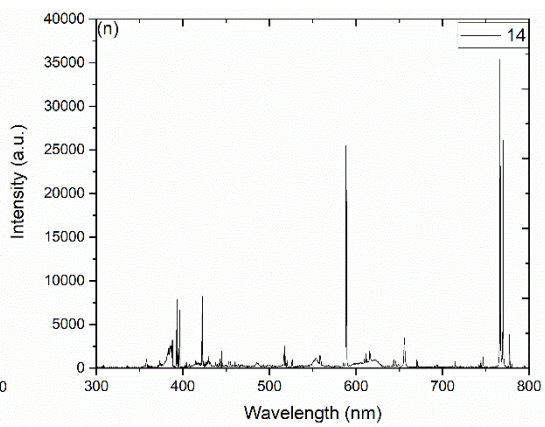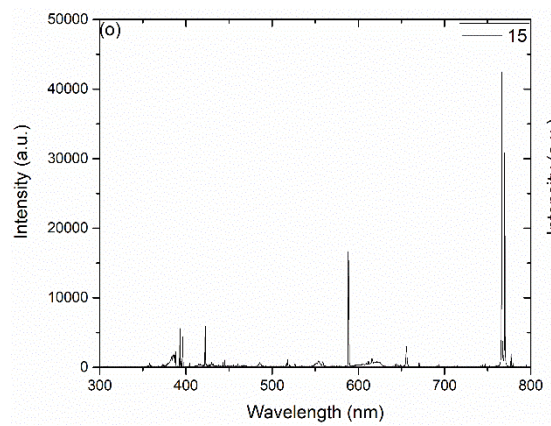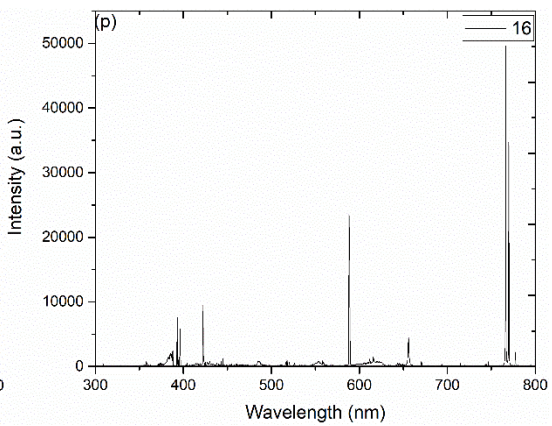

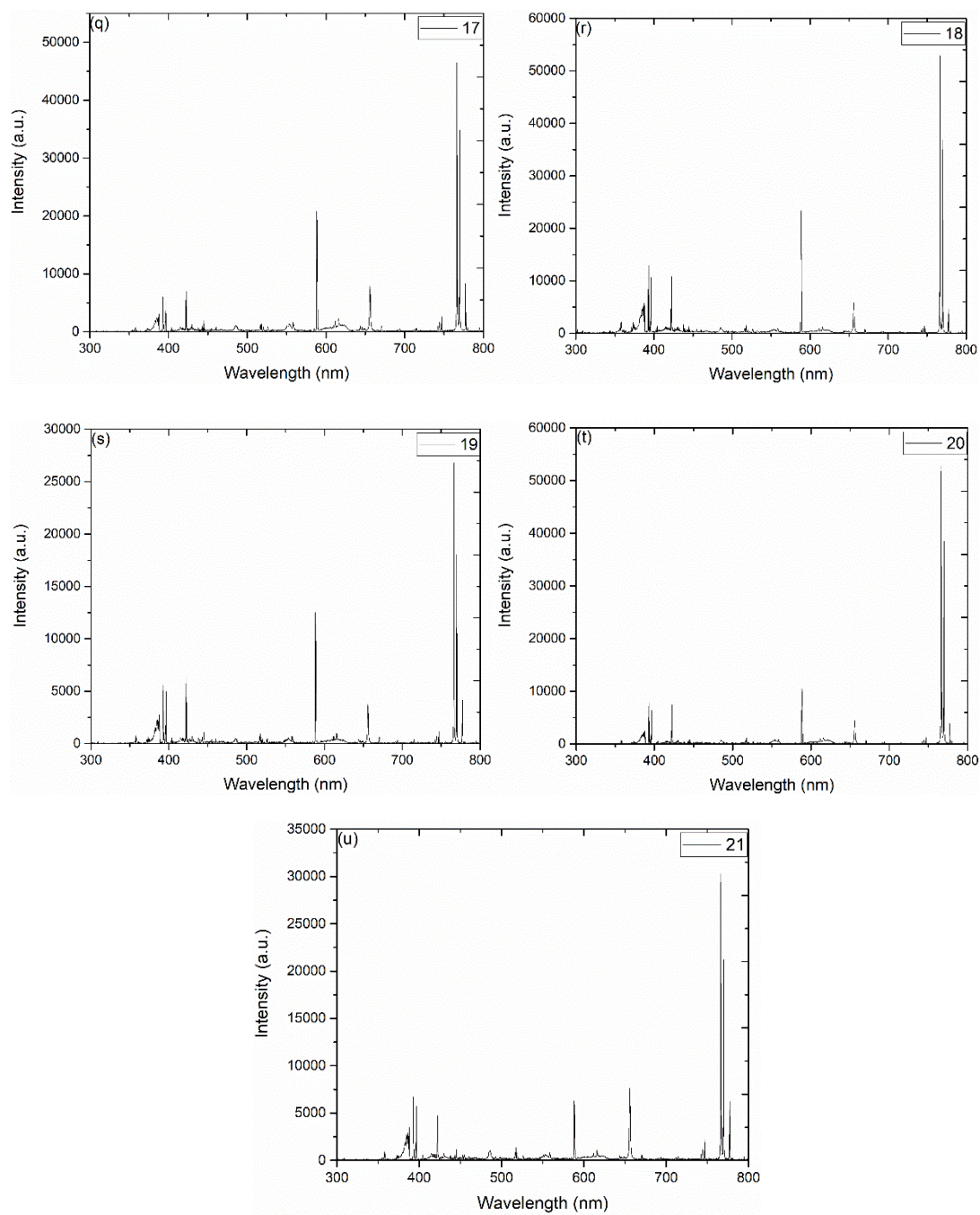

**Figure S1.** Typical LIBS spectra of 21 experimental samples with different doping levels.

Supplement: Supplementary file 1 [file foods-12-01710-s001.zip › foods-2312354-supplementary.pdf]
